# Overexpression of lncRNA LINC00665 inhibits the proliferation and chondroblast differentiation of bone marrow mesenchymal stem cells by targeting miR-214-3p

**DOI:** 10.1186/s13018-023-04475-0

**Published:** 2024-01-03

**Authors:** Siyuan Chen, Hui Liu, Yue Wang, Shuyuan Wang, Bo Yang, Di Sun, Pengxiao Sun

**Affiliations:** 1 Surgery of Spinal Degeneration and Deformity, Affiliated Hospital of Guangdong Medical University, Zhanjiang, 524000 China; 2 Department of Nursing, Peking University Third Hospital Qinhuangdao Hospital, Qinhuangdao, 066000 China; 3 Department of Orthopedics, Peking University Third Hospital Qinhuangdao Hospital, Qinhuangdao, 066000 China; 4 First Department of Joint, Xi’an International Medical Center Hospital, No.777, Xitai Road, Gaoxin District, Xi’an, 710000 China

**Keywords:** Osteoarthritis, lncRNA LINC00665, miR-214-3p, Bone marrow mesenchymal stem cell, Chondroblast differentiation

## Abstract

**Background:**

Osteoarthritis is a chronic disease mainly involving the damage of articular cartilage and the whole articular tissue, which is the main cause of disability in the elderly. To explore more effective treatment measures, this study analyzed the regulatory role and molecular mechanism of lncRNA LINC00665 (LINC00665) in the chondrogenic differentiation of bone marrow mesenchymal stem cells (BMSCs), providing a valuable theoretical basis for the pathogenesis and patient treatment of osteoarthritis.

**Methods:**

Osteoarthritis tissues and healthy tissues were obtained from 52 patients with osteoarthritis and 34 amputated patients without osteoarthritis, and the levels of LINC00665 and miR-214-3p were assessed by RT-qPCR. BMSCs were cultured and induced chondrogenic differentiation. The proliferation ability of BMSCs was detected by CCK-8 method, and the apoptosis level of BMSCs was evaluated by flow cytometry. The content of proteoglycan-glycosaminoglycan (GAG) in cartilage matrix was determined by Alcian blue staining. In addition, the binding relationship between LINC00665 and miR-214-3p was verified by luciferase reporter assay, and the molecular mechanism was further analyzed.

**Results:**

In osteoarthritis tissues, LINC00665 was elevated and miR-214-3p was down-regulated. With the chondrogenic differentiation of BMSCs, the level of GAG increased, and LINC00665 expression gradually decreased, while miR-214-3p level was on the contrary. After transfection of pcDNA3.1-LINC00665 in BMSCs, cell proliferation capacity was decreased, apoptosis rate was increased, and GAG content was reduced. Moreover, LINC00665 sponged miR-214-3p and negatively regulate its expression. Transfection of pcDNA3.1-LINC00665-miR-214-3p mimic changed the regulation of pcDNA3.1-LINC00665 on the viability and chondrogenic differentiation of BMSCs.

**Conclusions:**

Overexpression of lncRNA LINC00665 inhibited the proliferation and chondrogenic differentiation of BMSCs by targeting miR-214-3p. The LINC00665/miR-214-3p axis may improve joint damage and alleviate the progression of osteoarthritis.

## Background

Osteoarthritis is a slowly developing degenerative bone and joint disease, which is characterized by joint pain, stiffness and limited movement [1]. According to epidemiological data, there are 250 million cases of osteoarthritis worldwide, and most of them are middle-aged and elderly people [2, 3]. In the United States, more than 13.8% of the population is affected by osteoarthritis, while osteoarthritis appears to affect more adults in China, with a reported prevalence of osteoarthritis about 46.3% nationwide [4, 5]. The occurrence of osteoarthritis is not caused by a single factor, but the result of the combined action of many factors, which is generally believed to be related to obesity, joint injury and age of patients [6]. Bone marrow mesenchymal cells (BMSCs) are adult stem cells, which are derived from mammalian bone marrow matrix and mainly exist in connective tissues and organ stroma. BMSCs have multi-directional differentiation potential, regeneration ability, hematopoietic support and repair functions [7]. BMSCs have been used in the treatment of osteoarthritis based on their powerful amplification ability and good anti-inflammatory effect [8]. For example, a cell-based approach to establish the bilayered constructs to promote cartilage regeneration was proposed as early as 2012 [9]. However, current clinical application of means can only improve the symptoms of osteoarthritis to a certain extent to relieve pain, whereas cannot effectively cure it. Therefore, it is particularly critical to systematically elaborate the pathological mechanism of osteoarthritis and explore new treatment methods.

Long noncoding RNAs (lncRNAs) have attracted a lot of attention and research in recent decades, and countless evidence have revealed the regulatory ability of lncRNAs in different diseases. Inevitably, the involvement of abnormally expressed lncRNAs in the diagnosis and treatment of osteoarthritis, and influence on its pathogenesis has also been documented [10]. As early as 2019, Zhang et al. proposed that lncRNA MALAT1 affects cell growth and promotes the development of osteoarthritis by regulating the miR-150-5p/AKT3 axis [11]. LncRNA LINC00665 was first discovered to be located on chromosome 19q13.12 in 2018 and is commonly expressed abnormally in cancer [12]. For example, LINC00665 was highly expressed in ovarian cancer, and LINC00665 knockdown attenuated cell viability and favored patient prognostic survival by targeting miR-34a-5p [13]. Intriguingly, LINC00665 sponge miR-122-3p mediated the progression of rheumatoid arthritis [14]. Therefore, we hypothesized that LINC00665 may be a potential target for osteoarthritis.

This article examined the LINC00665 level in osteoarthritis tissue samples, and focused on the changes of LINC00665 and miR-214-3p levels during the chondrogenic differentiation of BMSCs. The impact of LINC00665 on the proliferation and apoptosis of BMSCs was analyzed by transfection of LINC00665 overexpression, and the exploration of the molecular mechanism of LINC00665 in osteoarthritis provided a new direction for the rehabilitation of patients.

## Methods

### Enrolled patients

The 52 patients with osteoarthritis who underwent knee joint replacement in Peking University Third Hospital Qinhuangdao Hospital were selected, and osteoarthritis cartilage tissues were isolated and obtained after surgery (*n* = 52), and normal cartilage tissues were obtained from 34 patients without osteoarthritis who underwent amputation during the same period (*n* = 34). The achieved tissue samples were properly stored in an ultra-low temperature freezer after the patient's surgery for subsequent use. The patients with osteoarthritis met the diagnostic criteria of osteoarthritis by the World Health Organization, and none of the participants had tumors, immune system diseases or cardiovascular diseases.

### Ethical approval

The studies involved were all in accordance with the relevant guidelines of the Declaration of Helsinki and were approved by the Medical Ethics Committee of Peking University Third Hospital Qinhuangdao Hospital. Meanwhile, the enrolled patients signed written consent materials on the premise of informed consent.

### Culture and chondrogenic differentiation of BMSC

BMSCs were derived from ATCC, which were inoculated in DMEM medium supplemented with FBS and penicillin–streptomycin (Invitrogen, USA) at a ratio of 10% and 1%, respectively, and cultured at 37 °C in an incubator with 5% CO_2_.

BMSCs chondrogenesis differentiation was induced with the assistance of the StemPro® Chondrogenesis Differentiation Kit (Thermo Scientific, USA) and performed for 0, 7, and 14 days. The cartilage matrix proteoglycan glycosaminoglycan (GAG) content at 620 nm was detected by Alcian blue staining kit (GENMED, USA).

### Transfection of BMSC

The pcDNA3.1-LINC0066, pcDNA3.1-LINC00665-mimic NC and pcDNA3.1-LINC00665-miR-214-3p mimic synthesized by GenePharma (Shanghai, China) were transfected into BMSCs respectively with the presence of Lipofectamine 2000 (Invitrogen, USA) reagent. Transfected cells were harvested after 48 h of incubation in 6-well plates and the transfection efficiency was measured.

### BMSC proliferation and apoptosis assay

BMSCs were transferred to 96-well plates and supplemented with CCK-8 reagent (Dojindo, Japan) at 0,1,2,3, and 4 days after inoculation. After adding CCK-8 reagent and continuing culture for 2 h, the OD value at 450 nm was measured using a microplate reader.

BMSCs were washed with PBS and collected by centrifugation, and then incubated with ANNEXIN V-FITC/PI kit (Solebo, Beijing) for 1 h. Finally, the apoptosis of cells was detected and analyzed by flow cytometry.

### Real-time quantitative PCR (RT-qPCR) assay

The process of total RNA extraction from sample tissues and BMSCs utilized TRIzol reagent. And cDNA was obtained by PrimeScript RT Master Mix Kit (Takara, Japan) after quality testing of RNA. In addition, the RT-qPCR reaction system was configured with the participation of SYBR Green qPCR Master Mix Kit (Takara, Japan) and detected by ABI 7500 system (Applied Biosystems, USA). GAPDH and U6 were used as internal standards for LINC00665 and miR-214-3p, and their relative levels were calculated according to the 2^−ΔΔCt^ method.

### Dual-luciferase reporter assay

The binding sites of LINC00665 and miR-214-3p were inserted into the pGL3 vector (Promega, USA) to construct WT-LINC00665, and mutated to obtain MUT-LINC00665. WT-LINC00665 or MUT-LINC00665 was co-transfected into BMSCs with mimic NC or miR-214-3p mimic using Lipofectamine 2000. After 48 h, the luciferase activity was evaluated by Dual-Luciferase Reporter Assay Kit (Promega, USA).

### Statistical analysis

The obtained data were processed by GraphPad Prism 7.0 software and expressed as mean ± standard deviation (SD). Statistical differences between groups were analyzed by t-test and one-way ANOVA with post-hoc Tukey test. *P* < 0.05 was considered significant.

## Results

### Basic clinical data of the participants

The basic clinical data of the participants were recorded in Table 1. The levels of ESR, CRP, WBC and Neutrophils count were increased in patients with osteoarthritis compared with healthy controls (*P* < 0.05).

**Table 1:** Basic clinical information for patients with osteoarthritis and healthy individuals

Variables	Participants		*P* value
	Healthy (n = 34)	Osteoarthritis (n = 52)	
Age (years)	50.69 ± 15.54	51.61 ± 9.69	0.717
Gender (Male/Famale)	15/19	19/33	0.533
BMI (kg/m^2^)	24.89 ± 5.53	25.14 ± 4.94	0.829
ESR (mm/h)	6.19 ± 1.46	20.68 ± 4.08	< 0.001
CRP (mg/L)	4.59 ± 1.57	19.45 ± 4.41	< 0.001
WBC (10^9^/L)	5.11 ± 1.37	12.32 ± 3.26	< 0.001
Neutrophils count (10^9^/L)	4.20 ± 1.45	5.03 ± 1.75	0.023
Lymphocytes count (10^9^/L)	2.78 ± 0.95	2.57 ± 0.82	0.281

BMI, body mass index; ESR, erythrocyte sedimentation rate; CRP, C-reactive protein; WBC, white blood cell count. Mean ± standard deviation

### LINC00665 level was elevated in osteoarthritic tissue

**Fig. 1:**
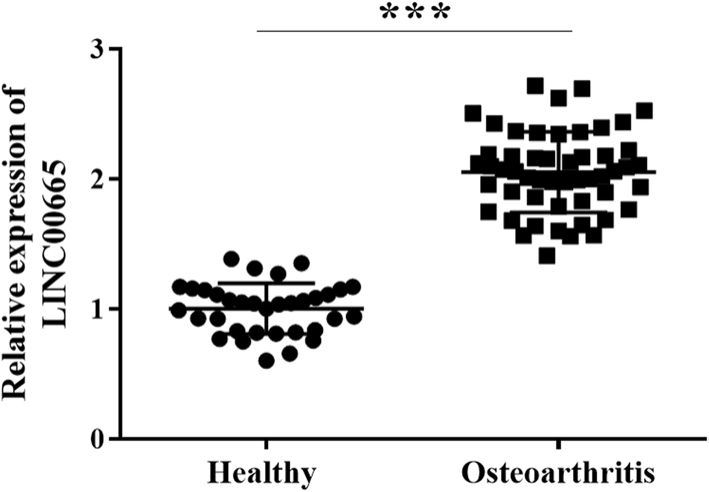
LINC00665 was elevated in osteoarthritis tissues, as detected by RT-qPCR in 34 healthy tissues and 52 osteoarthritis tissues. (***P < 0.001, compared with healthy tissue)

Healthy tissues and osteoarthritis tissues were obtained from the included patient samples and LINC00665 levels were detected by RT-qPCR. In Fig. 1, LINC00665 was eminently elevated in osteoarthritic tissues compared with the healthy tissues.

### Chondrogenic differentiation and activity regulation of BMSC

**Fig. 2:**
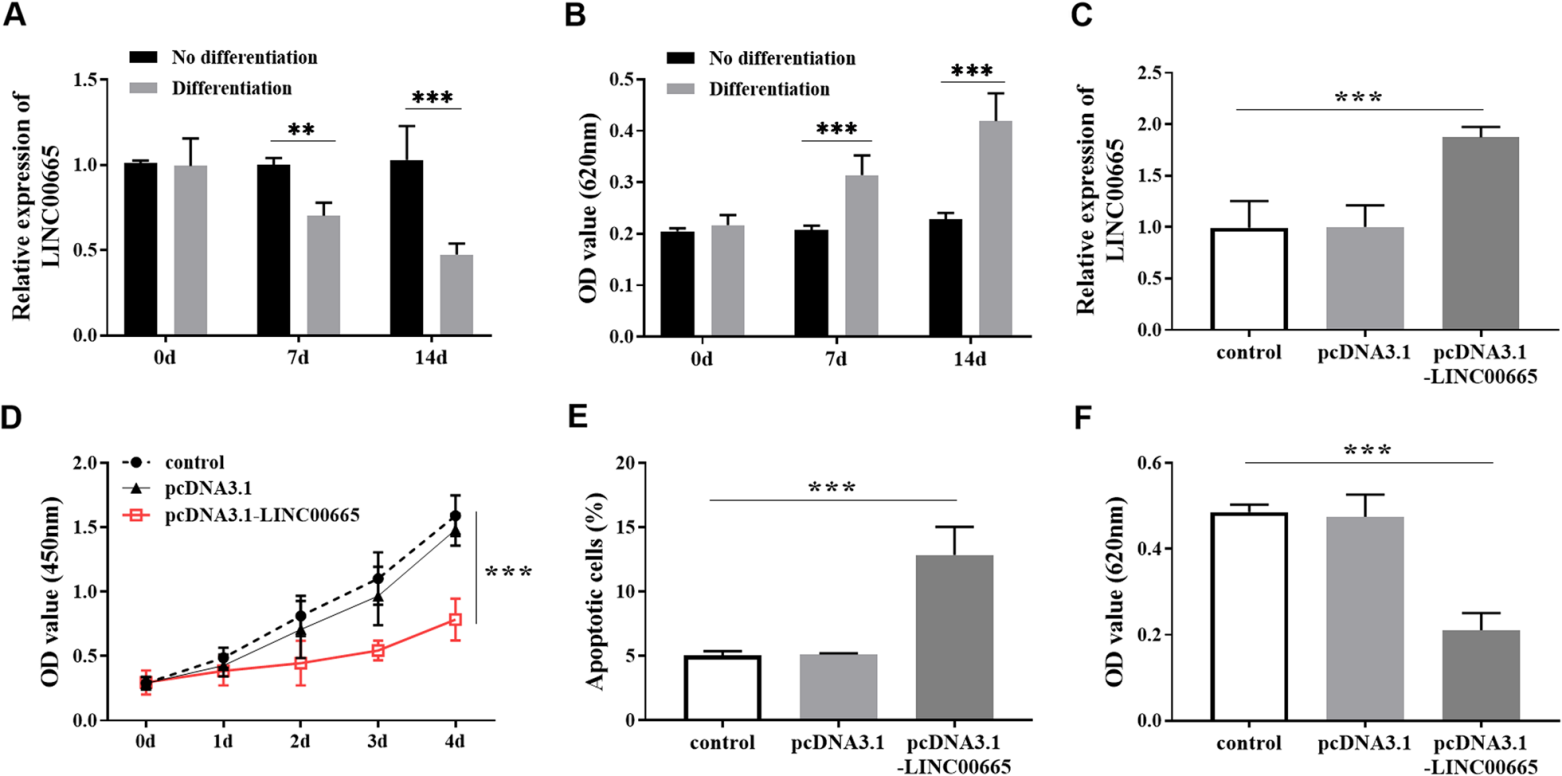
Chondrogenic differentiation and activity regulation of BMSCs. **A**. LINC00665 level in BMSCs chondrogenic differentiation within 14 days. **B**. The change in cartilage matrix proteoglycan GAG level within 14 days of BMSCs chondrogenic differentiation was detected by Alcian blue staining. (** *P* < 0.01, *** *P* < 0.001, compared with no differentiation group)** C**. Transfection efficiency of LINC00665 overexpression in BMSCs. **D**. LINC00665 overexpression inhibited the proliferation of BMSCs. **E.** LINC00665 overexpression increased the apoptosis rate of BMSCs. **F.** LINC00665 overexpression reduced the chondrogenic differentiation of BMSCs. (***P < 0.001, compared with the control group)

BMSCs were cultured and induced, and the relative LINC00665 levels before and after cell differentiation were detected via RT-qPCR. The relative expression of cartilage matrix proteoglycan GAG was detected by Alcian blue staining at 620 nm (0d, 7d, and 14d). The results showed that the LINC00665 level in BMSCs decreased gradually after chondrogenic differentiation (Fig. 2A). We hypothesized that LINC00665 may play a role in the chondrogenic differentiation of BMSCs. Fig. 2B illustrated the gradual increased in GAG content with increasing induction time. To further explore the regulatory function of LINC00665 in osteoarthritis, pcDNA3.1-LINC00665 was transfected into BMSCs to construct LINC00665 overexpression, and the transfection efficiency is shown in Fig. 2C. Cell viability analysis showed that pcDNA3.1-LINC00665 inhibited the proliferation level of BMSCs (Fig. 2D) and promoted the apoptosis of BMSCs (Fig. 2E). In addition, the GAG level was decreased after transfection with pcDNA3.1-LINC00665 in Fig. 2F. Therefore, it was speculated that high expression of LINC00665 inhibited the cell viability and chondrogenic differentiation of BMSCs.

### The binding relationship between LINC00665 and miR-214-3p

**Fig. 3:**
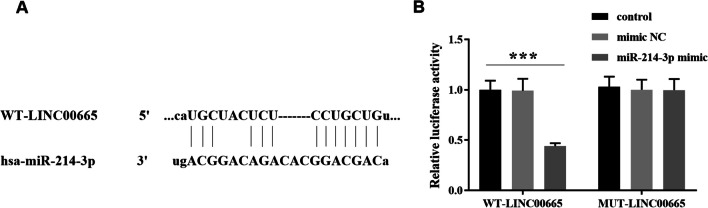
The study on relationship between LINC00665 and miR-214-3p. **A**. Binding sites exist for LINC00665 and miR-214-3p. **B**. Luciferase reporter assay verified the LINC00665 sponge miR-214-3p. (***P < 0.001, compared with the control group)

As shown in Fig. 3A, binding sites exist between LINC00665 and miR-214-3p. Then, we verified the targeting relationship between LINC00665 and miR-214-3p by luciferase reporter assay. The results of Fig. 3B showed that co-transfection of WT-LINC00665 and miR-214-3p mimic reduced the luciferase activity of BMSCs.

### The expression level of miR-214-3p

**Fig. 4:**
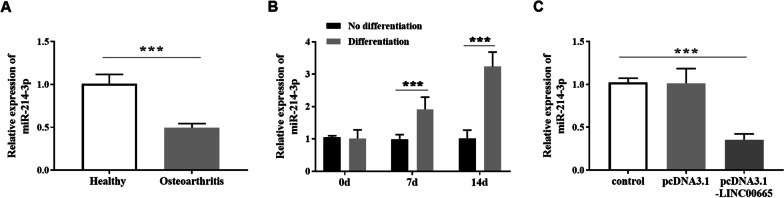
Expression of miR-214-3p in osteoarthritis tissues and BMSCs.** A**. miR-214-3p in osteoarthritis and healthy tissues. (***P < 0.001, compared with healthy tissue) **B**. miR-214-3p level was gradually up-regulated in chondrogenic differentiation of BMSCs. (*** *P* < 0.001, compared with no differentiation group)** C**. The expression of miR-214-3p was decreased after transfection of pcDNA3.1-LINC00665 in BMSCs. (***P < 0.001, compared with the control group)

Based on the fact that miR-214-3p is a target of LINC00665, the miR-214-3p level in osteoarthritis was revealed. Figure 4A displayed the low miR-214-3p level in osteoarthritis tissues. In the process of chondrogenic differentiation of BMSCs, miR-214-3p was up-regulated with the extension of induction time (Fig. 4B). Moreover, miR-214-3p was relatively decreased after transfection of pcDNA3.1-LINC00665 in BMSCs was shown in Fig. 4C. These results indicated that LINC00665 negatively regulated the expression of miR-214-3p.

### miR-214-3p mimic reversed the effect of pcDNA3.1-LINC00665 on BMSC

**Fig. 5:**
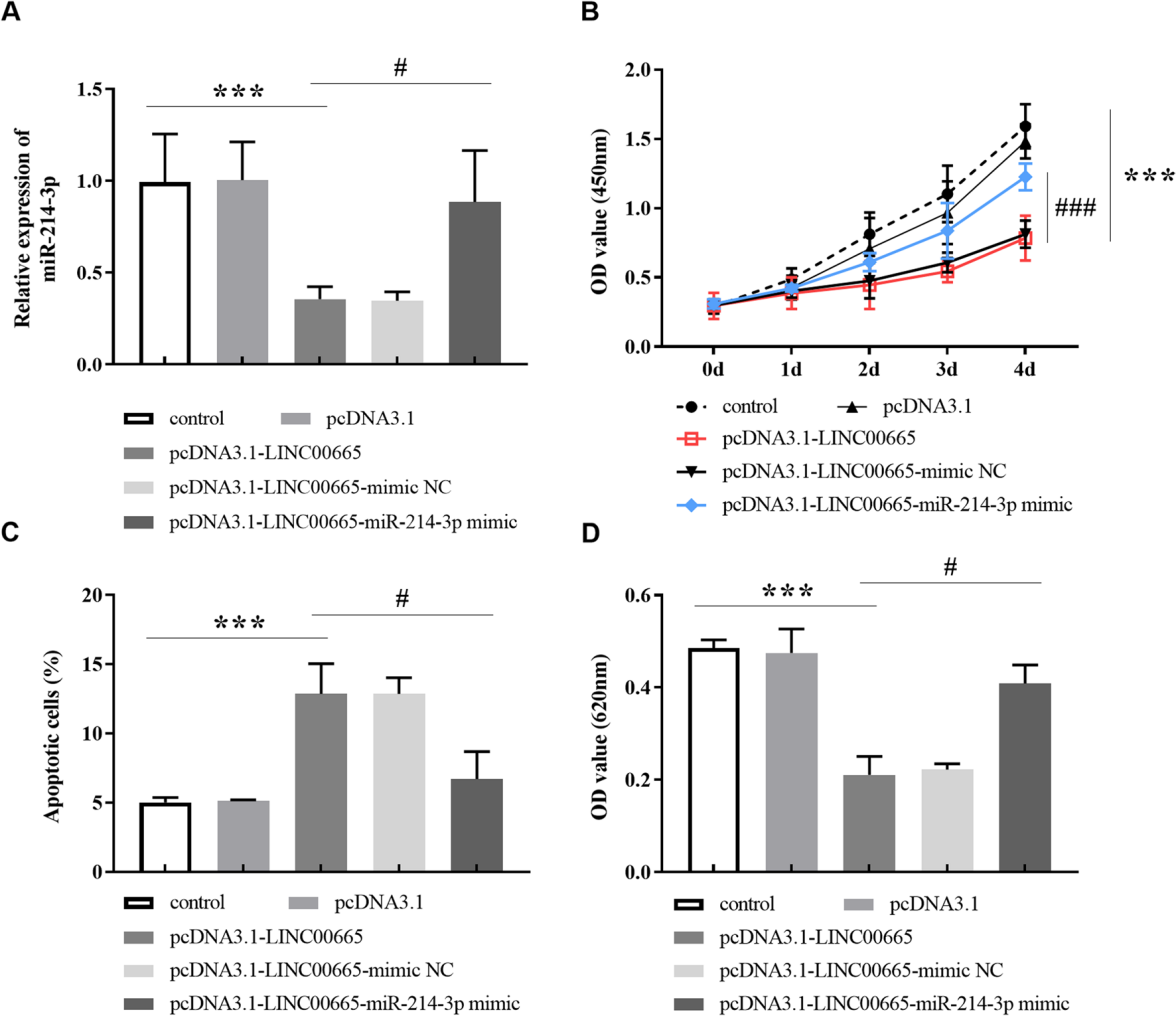
Transfection of pcDNA3.1-LINC00665-miR-214-3p mimic reversed the effect of LINC00665 overexpression on BMSCs.** A**. Relative expression of miR-214-3p in BMSCs after different transfection.** B**. The inhibitory effect of pcDNA3.1-LINC00665 on the proliferation of BMSCs was restored after transfection with pcDNA3.1-LINC00665-miR-214-3p mimic using CCK-8 method. **C**. The promoting function of pcDNA3.1-LINC00665 on the apoptosis rate of BMSCs was recovered after transfection with pcDNA3.1-LINC00665-miR-214-3p mimic according to flow cytometry detection.** D**. GAG content in BMSCs was increased after transfection of pcDNA3.1-LINC00665-miR-214-3p mimic. (***P < 0.001, compared with the control group. ^#^*P* < 0.05, ^###^*P* < 0.001, compared to the pcDNA3.1-LINC00665)

Furthermore, we transfected pcDNA3.1-LINC00665-mimic NC and pcDNA3.1-LINC00665-miR-214-3p mimic to investigate the regulation of LINC00665 on BMSCs by targeting miR-214-3p. In Fig. 5A, miR-214-3p levels were up-regulated after transfection with pcDNA3.1-LINC00665-miR-214-3p mimic, compared with pcDNA3.1-LINC00665. In addition, miR-214-3p mimic altered the inhibitory ability of pcDNA3.1-LINC00665 on the proliferation of BMSCs (Fig. 5B), while reduced the apoptosis rate of BMSCs (Fig. 5C). After measuring GAG content in BMSCs in Fig. 5D, it was also found that transfected pcDNA3.1-LINC00665-miR-214-3p mimic could up-regulate GAG content compared with pcDNA3.1-LINC00665.

## Discussion

According to the cause of the disease, osteoarthritis can be divided into two categories, one is primary osteoarthritis and the other is secondary osteoarthritis [15]. The former is thought to be caused by systemic or local factors, mainly related to genetic and physical factors. The latter occurs on the basis of primary diseases, such as joint injuries, metabolic diseases, and congenital joint malformations [16, 17]. The symptoms of patients with osteoarthritis are joint pain and limited joint movement, so the key to treatment is to relieve the pain of patients, restore joint function, and delay the progression of the disease [18]. Studies have noted that BMSCs may be involved in the treatment of osteoarthritis through their tissue repair function and anti-inflammatory properties [19]. Zheng et al. [20] proposed that osteoarthritis is closely related to osteogenic differentiation of BMSCs, and ROCK1 signaling in BMSCs can assist in mediating the process of osteoarthritis. He et al. [21] indicated that BMSCs-derived exosomes can restore cartilage damage and relieve pain in osteoarthritis. Yang and colleagues also elucidated the molecular mechanism of chondrogenic differentiation of BMSCs and its potential in tumor therapy [22]. Based on the above discussion, we further explored BMSCs and osteoarthritis by mediating LINC00665.

By analyzing the clinical data of patients with osteoarthritis, it can be noted that there was no significant difference in age, gender, BMI and lymphocytes count levels between patients and healthy people, while the ESR, CRP, WBC and Neutrophils count were elevated. Existing evidence suggested that age and BMI are risk factors for the development of osteoarthritis [23], and the results of this study may be related to the small sample number. Furthermore, the up-regulated LINC00665 in osteoarthritis was found in this study. As a concerned lncRNA associated with human disease, LINC00665 was reported to increase dramatically in cell models of cerebral ischemia–reperfusion injury [24], and it was also enhanced in numerous tumors such as breast cancer and prostate cancer [25]. Deng et al. [26] found that LINC00665 was elevated in rats with spinal cord injury, and inhibition of its expression was beneficial to cell survival and control inflammatory response. Wang et al. [14] revealed the LINC00665 sponge miR-122-3p/EIF2AK1 axis to affect cell viability, thereby suppressing the deterioration of rheumatoid arthritis. Therefore, we speculated that LINC00665 knockdown may improve joint injury and alleviate the progression of osteoarthritis, while LINC00665 overexpression may aggravate the condition. To understand the mechanism by which LINC00665 regulates BMSCs to treat osteoarthritis, we performed in vitro cellular experiments. This confirmed that LINC00665 level gradually decreased during the chondrogenic differentiation of BMSCs. After transfection of pcDNA3.1-LINC00665, the proliferation ability of BMSCs was decreased and the apoptosis rate was increased, while the differentiation degree of BMSCs was inhibited. Similarly, Ji et al. [27] suggested that BMSCs proliferation was prevented when lncRNA BLACAT1 was elevated. And, lncRNA XIST was enhanced in osteoarthritis tissues, and XIST decreased with chondrogenic differentiation of BMSCs. Overexpression of XIST can affect the occurrence of chondrogenic differentiation [28].

In existing studies, miR-214-3p was reported to be associated with the occurrence of osteoarthritis. miR-214-3p level was low in osteoarthritis chondrocytes, and miR-214-3p had a protective function in cartilage metabolism [29]. miR-214-3p was down-regulated in osteoarthritis, and silencing miR-214-3p aggravated osteoarthritis by activating the NF-κB signaling pathway [30]. In addition, lncRNA PVT1 directly targeted miR-214-3p in HCC, and downregulation of miR-214-3p was negatively correlated with PVT1 levels [31]. The low expression of miR-214-3p in Parkinson's disease, and lncRNA SNHG14 sponge miR-214-3p interfered with the cure of patients [32]. Consistent with previous data, we verified that miR-214-3p was a downstream target of LINC00665 and its level was reduced in osteoarthritis tissues. Contrary to the content of LINC00665, miR-214-3p increased with the progress of BMSCs chondrogenic differentiation. Transfection of pcDNA3.1-LINC00665-miR-214-3p mimic restored the inhibition of LINC00665 overexpression on proliferation of BMSCs and reversed the promotion of apoptosis of BMSCs induced by LINC00665 overexpression. In the investigation of chondrocyte inflammation, miR-214-3p mimic also had the effect of accelerating chondrocyte proliferation and reducing cell death [33]. Upregulation of miR-214-3p can ameliorate cartilage and synovial membrane damage in osteoarthritis mice and mediate cell activity by targeting downstream proteins [34].

## Conclusions

Taken together, the empirical experiments and analysis of this study revealed that LINC00665 was elevated in osteoarthritis, while miR-214-3p was decreased. LINC00665 overexpression inhibited the proliferation and promoted the apoptosis of BMSCs by targeting miR-214-3p, which regulated the differentiation of BMSCs. Therefore, LINC00665 may serve as a key target for osteoarthritis therapy and amelioration of joint injury according to LINC00665/miR-214-3p axis.

## Data Availability

All data generated or analyzed during this study are included in this article. Further enquiries can be directed to the corresponding author.

## Abbreviations

BMSCs: Bone marrow mesenchymal stem cells
lncRNAs: Long noncoding RNAs
SD: Standard deviation
